# Current status of contact lenses usage in Korea: A population-based cohort study 2021

**DOI:** 10.1371/journal.pone.0296279

**Published:** 2024-03-20

**Authors:** Minsun Kim, Ji-Sun Paik, Daran Kim, Ho Sik Hwang, Kyungdo Han, Kyung-Sun Na

**Affiliations:** 1 Department of Ophthalmology, College of Medicine, The Catholic University of Korea, Seoul, Republic of Korea; 2 Department of Statistics and Actuarial Science, Soongsil University, Seoul, Republic of Korea; University of New South Wales, AUSTRALIA

## Abstract

**Purpose:**

To investigate trends in contact lens usage in a nationally representative sample of the Korean population in 2021.

**Methods:**

For this retrospective study, we analyzed data of 3,601 Korean participants aged 10–59 years, from the Korea National Health and Nutrition Examination Survey (KNHANES 2021 version), who underwent eye examination, of whom 1,136 individuals (274 men and 862 women) were contact lens users. The demographic trend among Korean contact lens wearers was examined using statistical analyses to investigate the changes in their contact lens-wearing experience, duration of lens use, type of lens used, location of purchase, presence of an Eye Care Practitioner(ECP)’s prescription, lens-related ophthalmic complications, and type of lenses worn at the time of complications, according to sex. Multivariable logistic regression analysis was conducted to examine the association of each variable with the rate of complications and use of soft lenses.

**Results:**

The average age of the contact lens users was 33.42±0.33 years, with 70.36% (weighted percentage) of users being women who used contact lenses for significantly longer periods than men (*p*<0.001). Additionally, only wearing of cosmetic lenses was significantly correlated with the occurrence of complications (*p* = 0.006), and 6.76% of users purchased lenses without a prescription. Multivariate analysis among the contact lens users revealed a significant relationship between the complication rate and female sex (*p* = 0.002), pre-existing eye disease diagnosed by ECPs (*p* = 0.0288), and duration of contact lens use (*p*<0.0001).

**Conclusion:**

We identified sex differences in contact lens usage trends in Korea. The main changes observed were an increase in middle-aged lens users and a decrease in female users compared to that in the early 2000s. In addition, contact lens complications were significantly associated with sex and pre-existing eye disease. Therefore, those wearing contact lenses for extended periods should exercise caution and consult eye care specialists in the presence of any symptoms.

## Introduction

Since the invention of contact lenses as an alternative to glasses in 1888 by Adolf Eugen Fick [1], significant advances have been made in the material and design of contact lenses. Many people utilize contact lenses today to address refractive errors and for cosmetic reasons [2–4]. The design, material, and manufacturer of lenses that are easily accessible in each nation are all related to actual trends in contact lens wear. In addition, the distribution of refractive errors, the level of training of contact lens dispensers (optometrists, ECPs, or unregulated lens sellers), the age and sex ratio of the population, and the socioeconomic conditions of the patients affect the status of prescriptions for contact lenses [5].

With the increasing incidence of myopia at younger ages [6], the use of contact lenses for myopia correction is becoming common. In addition, the age at which corrective lens usage is suggested is also reported to be decreasing, especially because of recent advances in slowing the progression of myopia, either with overnight orthokeratology (OK) or use of multifocal soft contact lenses [5, 7–10].

The yearly international contact lens prescribing report is the most extensive annual multinational study of contact lens prescriptions; it reports the results of large-scale surveys in 20 countries over 20 years, starting with a 1996 UK contact lens survey [5, 11]. However, it is regrettable that although the target countries have differences in contact lens prescription systems, these inter-country differences are not considered. Moreover, there may be a bias because contact lens practitioners in each country were only included in the dataset if they voluntarily responded to the survey. Although information on contact lens prescribing trends would be useful to facilitate national eye-care planning, there are only a few population-based or large-scale studies on prescribing status, especially in East Asia [12–15]. The concerns from a survey conducted by the Korean Contact Lens Society in 2008, is the decrease in age of contact lens users and rapid increase of cosmetic colored lens-related complications [16]. The Korea National Health and Nutrition Examination Survey (KNHANES) is an ongoing nationwide, population-based, cross-sectional health examination and survey that accumulates data on the health and nutritional status of the non-institutionalized population of South Korea. Ophthalmologic examinations were included in the survey in the latter half of 2008 to investigate the prevalence and risk factors of common eye diseases [17]. This study aimed to investigate contact lens prescribing trends using data collected from a recent large-scale Korean population-based survey of contact lens prescription status and complication history. In addition, we analyzed the characteristics of individuals who experienced contact lens complications. South Korea operates within a distinctive context where the conventional concept of an "optometrist" is notably absent. The term "Eye Care Practitioner (ECP)" was used to prevent confusion of readers, because it encompass both ophthalmologists and optometrists who is capable of examining eyes and recognizing and managing eye diseases.

## Methods

### Study population

The KNHANES is a national surveillance system that has been assessing the health and nutritional status of Koreans since 1998. Contact lens-related investigations were conducted among participants aged >40 years in 2019 and 2020 and those aged 10–59 years in 2021. Since the present study requires investigation of various age groups, the 2021 data were utilized. Of the 7,090 individuals surveyed in the KNHANES in 2021, 3,603 individuals aged 10–59 years old were subjected to an eye examination, and 3,601 were included in this study; two participants were excluded due to being in an anophthalmia state or having undergone ocular enucleation or exenteration. In total, there were 1,136 participants who were using contact lenses in this study. The KNHANES data are publicly available (https://knhanes.kdca.go.kr/knhanes/sub03/sub03_02_05.do); the authors did not have access to information that could identify individual participants during or after data collection. This study was approved by the Institutional Review Board of the Catholic University of Korea, and the requirement for informed consent was waived because of the retrospective cohort nature of the study (IRB number: SC23ZISE0053).

### KNHANES survey for contact lens use

The KNHANES surveys are conducted annually using a rolling sampling design that involves a complex, stratified, multistage, probability-cluster survey of a representative sample of the noninstitutionalized civilian population in South Korea. The KNHANES is composed of three component surveys: a health interview, a health examination, and a nutrition survey. Health interviews and examinations are conducted by trained medical staff and interviewers at mobile examination centers. After completion of the surveys, data are assembled and stratified based on socioeconomic status; health behaviors; quality of life; healthcare utilization; anthropometric measures; biochemical profiles using fasting blood serum and urine; measures for dental health, vision, hearing, and bone density; radiography results; food intake; and dietary behavior [18]. The survey items relevant to the present study included questions on the experience of wearing contact lenses, duration of lens use, type of lens used, place of purchase, presence of an ECP’s prescription, lens-related ophthalmic complications (including keratitis), and the type of lenses worn at the time of complications. The types of lenses were classified into soft, hard (RGP), OK, and cosmetic lenses, respectively. The presence or absence of ocular conditions was identified based on self-reported responses from participants. Responses were solicited by selecting from among items pertaining to glaucoma, cataracts, macular degeneration, retinal vascular occlusion, diabetic retinopathy, and dry eye syndrome. Complications were identified based on self-reported responses from participants. Participants were asked about their experience with contact lens-related “ophthalmic complications,” including corneal inflammation, and responded with “Yes” or “No.” Data on the types and severity of complications were not collected.

### Statistical analyses

All statistical analyses were performed using SAS version 9.4 (SAS Institute, Cary, NC, USA). The KNHNES utilizes a multi-stage clustered probability design. Individual-level weights are assigned to estimate the population by accounting for the complex survey design, survey non-response, and post-stratification. Therefore, all data statistics of this survey were presented as weighted percentages throughout the study [18]. The clinical and demographic characteristics of the study participants were presented as mean ± standard error (SE) for continuous variables and as numbers with weighted percentages (%) for categorical variables. The *p*-value for continuous variables was obtained using Student’s *t*-test, and the *p*-value for categorical variables was obtained using the Rao–Scatt chi-square test. Multivariable adjusted logistic regression analysis was conducted to examine the odds ratio (OR) and 95% confidence interval (CI) for the association of each variable with the lens complication rate and with soft lens use. Model 1 was unadjusted, and Model 2 was adjusted for age, sex, income, region, duration of contact lens use, place of purchase, whether the lenses were prescribed by an ECP, and whether the participants had a history of eye disease. The *p*-value was calculated using hierarchical multivariate logistic regression analyses, and statistical significance was set at *p* <0.05.

## Results

The general characteristics of the participants wearing contact lenses are summarized in Table 1. Regarding the duration of use, the group with 1–5 years of use accounted for the majority of the samples at 33.17%. Women had a significantly longer period of use than men (*p*<0.001), with 46.45% of women and only 17% of men reporting usage of lenses for a duration of ≥5 years. Among male contact lens users, nearly half of them (45.59%) used the lenses for ≤1 year. The most commonly used lens type was soft lens for both sexes, and women were significantly more likely to use cosmetic lenses compared to men (*p*<0.0001).

**Table 1:** General characteristics of the study participants who use contact lenses according to sex in the Korean population (n = 1136).

	Weighted %(SE)			
	Total	Sex		
		Men	Women	*p-*value
	(n = 1136)	(n = 274)	(n = 862)	
Age				0.1379
10–19 years	9.59 (0.93)	5.87 (1.28)	11.16 (1.22)	
20–29 years	30.85 (1.98)	32.26 (4.02)	30.25 (2.01)	
30–39 years	28.88 (1.91)	32.26 (3.75)	27.46 (1.71)	
40–49 years	21.15 (1.24)	20.89 (2.6)	21.26 (1.38)	
50–59 years	9.53 (1.02)	8.72 (1.98)	9.87 (1.12)	
Sex				
Men	29.64 (1.74)			
Women	70.36 (1.74)			
Duration of contact lens use				**<0.0001**
< 1 year	29.19 (1.45)	45.59 (3.32)	22.28 (1.53)	
1–5 years	33.17 (1.69)	37.7 (3.4)	31.27 (1.89)	
5–10 years	18.45 (1.3)	9.34 (1.89)	22.28 (1.57)	
10 years <	19.19 (1.33)	7.37 (1.69)	24.17 (1.74)	
Lens type (1-month data)				
No lens use in the past month	41.48 (2.05)	38.65 (3.47)	42.67 (2.29)	0.2982
Soft lenses	31.54 (1.99)	33.51 (3.41)	30.71 (2.11)	0.425
Hard lenses (RGP)	2.56 (0.53)	3.71 (1.18)	2.08 (0.59)	0.1737
Orthokeratology	0.81 (0.22)	0.8 (0.41)	0.82 (0.27)	0.9718
Cosmetic lens	9.74 (1.23)	1.45 (0.93)	13.23 (1.73)	**<0.0001**
Other	0.1 (0.1)	0.35 (0.35)	-	-
Place of purchase				**0.0042**
Eye clinics	6.76 (0.82)	7.22 (1.59)	6.56 (0.85)	
Eyeglass stores	83.84 (1.26)	88.61 (1.93)	81.83 (1.52)	
Places other than an eyeglass store	8.9 (1.11)	3.89 (1.32)	11.01 (1.44)	
Do not know	0.5 (0.22)	0.27 (0.27)	0.6 (0.3)	
Whether contact lenses are prescribed by an ECP				0.5529
Yes	21.01 (1.41)	18.72 (2.44)	21.98 (1.59)	
No	77.33 (1.56)	79.13 (2.8)	76.57 (1.69)	
Do not know	1.66 (0.48)	2.15 (1.35)	1.45 (0.44)	
Complication due to contact lens, Yes	23.01 (1.29)	10.68 (1.82)	28.2 (1.71)	**<0.0001**
Contact lens experience with respect to ophthalmic complications				
Do not know	0.9 (0.9)	6.53 (6.31)	-	-
Soft lenses	64.24 (3.52)	88.45 (7.05)	60.38 (3.95)	**0.0151**
Hard lenses (RGP)	6.35 (1.38)	5.02 (3.52)	6.56 (1.54)	0.72
Orthokeratology	1.66 (0.85)	-	1.92 (0.98)	-
Cosmetic lens	29.4 (3.6)	-	34.09 (3.99)	-

SE, standard error

Purchasing lenses from an eyeglass stores was the most predominant method of purchase for both sexes (88.61% of men and 81.83% of women). In the same context, only 21.01% of the population purchased lenses based on prescriptions from ECPs. It can be inferred from this that most lens users purchase lenses without consulting their ECPs. Additionally, in the population that experienced complications, the use of soft lenses was significantly higher among men, at 88.45% in men and 60.38% in women (*p* = 0.0151).

**Fig 1:**
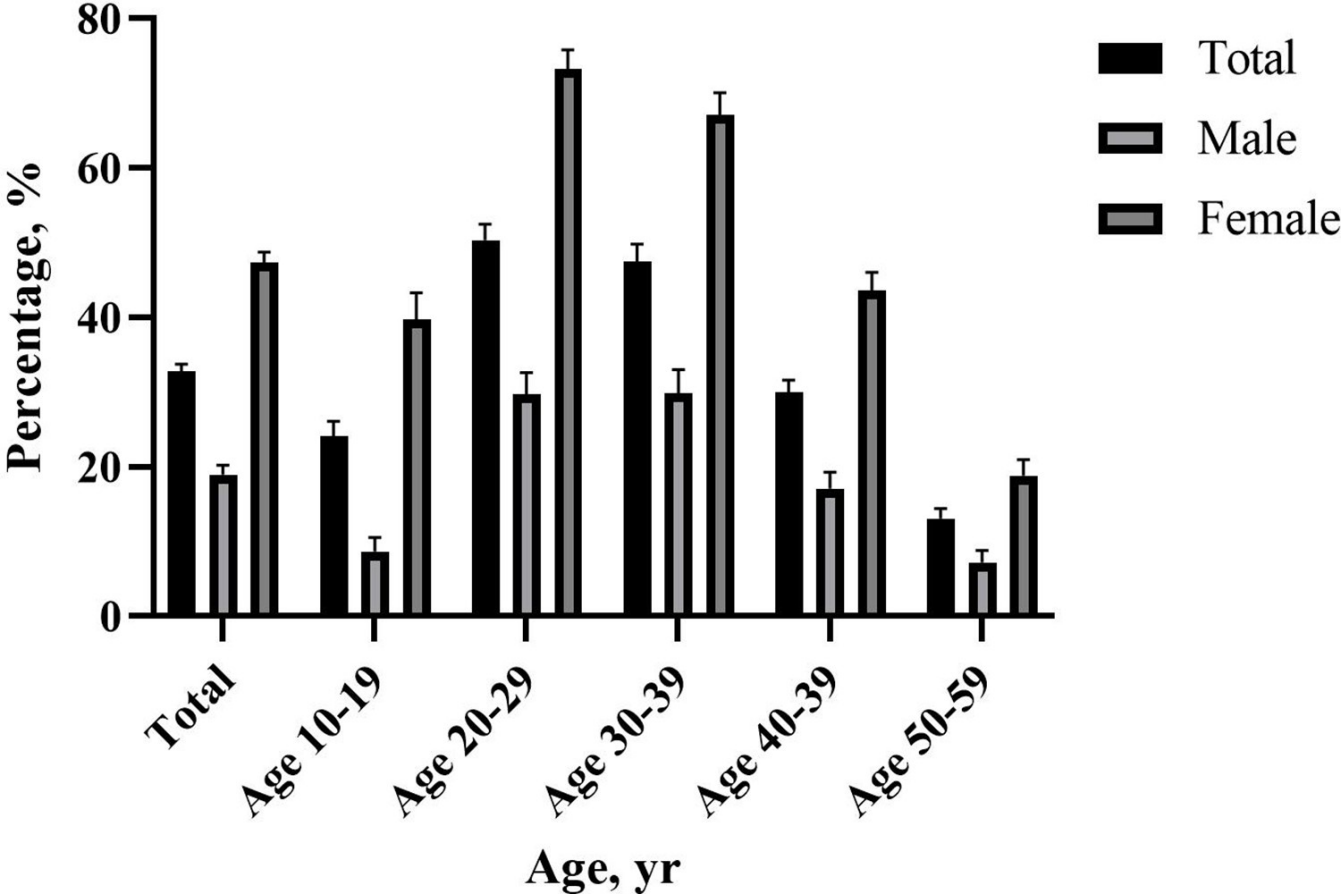
Prevalence of contact lens usage (%) according to sex.

The overall prevalence of contact lens use was 32.77%; in addition, only 18.96% of all men and 47.28% of all women had used lenses, indicating that women were the predominant lens wearers. The prevalence of contact lens use was highest in the 30–39-year age group for men and the 20–29-year age group for women (Fig 1). Among contact lens users, 70.36% (weighted percentage) of the participants were women. The mean age of participants with contact lens use was 33.42±0.33 years (*p*<0.0001).

**Fig 2:**
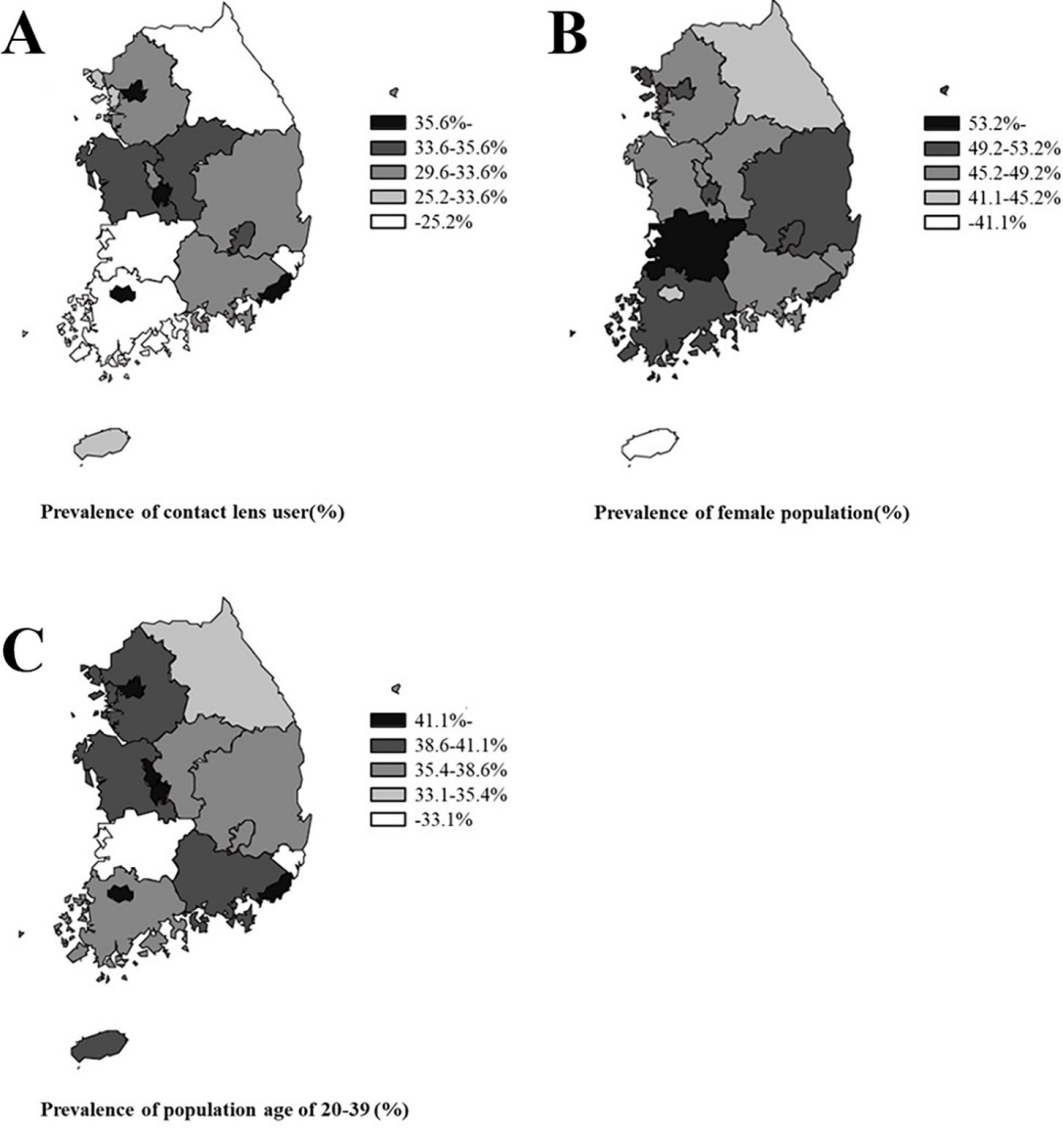
Prevalence of contact lens use in South Korea. (A) Overall prevalence of contact lens use in South Korea. (B) Prevalence of lens use among women in South Korea. (C) Prevalence of lens use in the 20–39-year age group in South Korea. *Reprinted from Statistical Geographic Information Service of Korea under a CC BY license, with permission from Lee Ju Won, Director of Geospatial Information Service Division(Statistics Korea), original copyright 2023.

**Fig 3:**
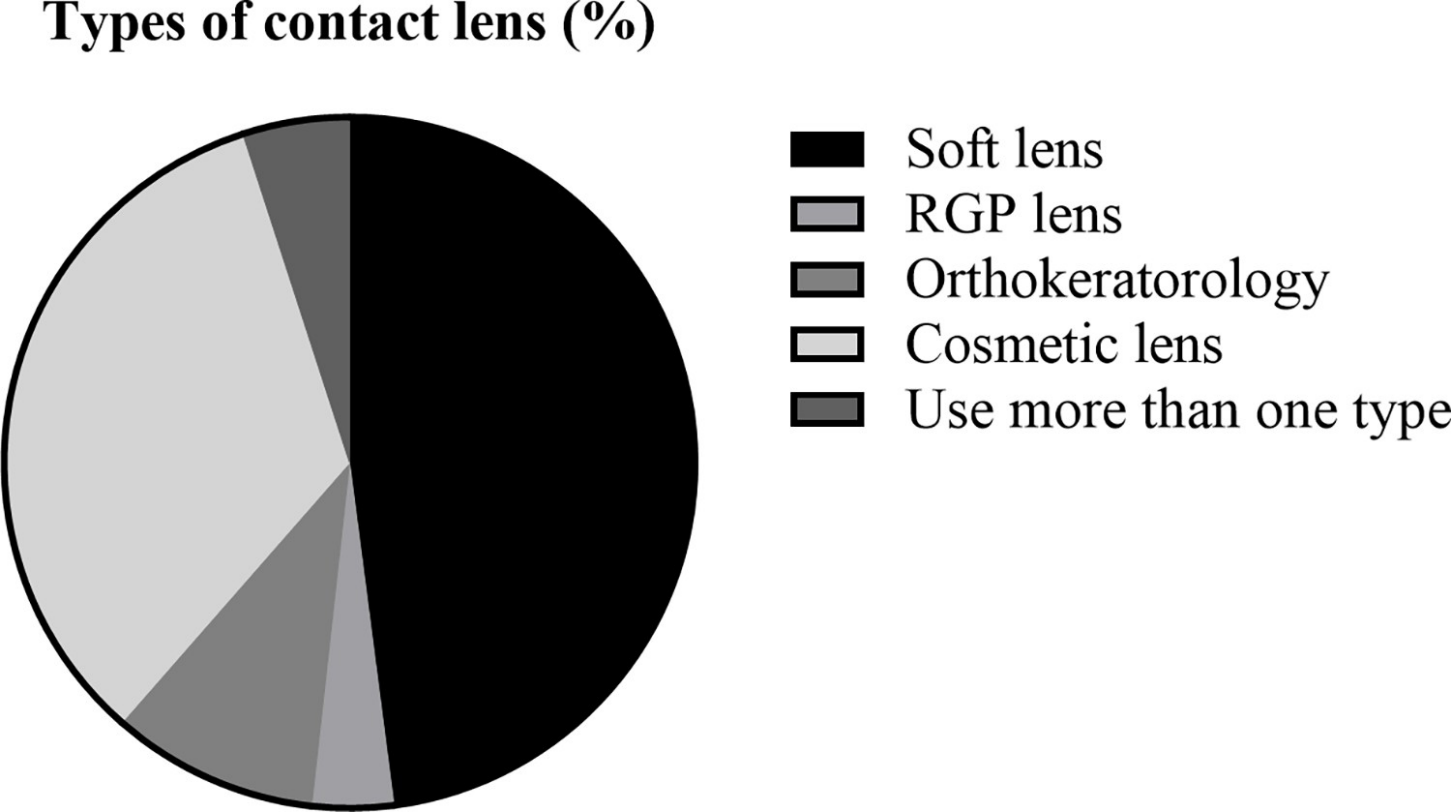
Prevalence of different types of contact lenses among contact lens users (%).

Fig 2 shows the prevalence of contact lens use in South Korea stratified according to various factors. To investigate whether urbanization, location of residence, population percentage of women, and age 20–39 years were associated with the prevalence of contact lens use, the percentage of contact lens users was stratified according to the population of city dwellers and other geographical areas. In addition, the percentages of women contact users and users aged 20–39 years were stratified into five groups (Fig 2B and 2C, respectively). Seoul, Gwangju, Busan, and Daejeon exhibited the highest usage rates. The lowest values were observed in Jeonnam and Ulsan. Soft lens use accounted for 48% of the total usage, followed by cosmetic lenses at 33%, corneal refractive lenses at 10%, two or more types at 5%, and hard lenses at 4% (Fig 3).

Table 2 presents a quantitative comparison of patients with contact lens complications. Although there was no statistically significant correlation between age and the frequency of complications, the frequency of complications was highest in the 30–39-year age group. The frequency of complications was also significantly higher among women compared to men (chi-square *p*<0.0001). In addition, there were some positive correlations between the duration of contact lens wear and the frequency of complications. Among the various types of contact lenses used, only cosmetic lens usage seemed to have a significant correlation with the occurrence of complications (*p* = 0.006). The mode of purchase and type of prescription did not appear to be significantly correlated with the occurrence of complications. Finally, soft lenses were associated with the highest rate of complications, possibly because they were the most commonly used type.

**Table 2:** Comparison of quantitative variables in patients with and without complications after using contact lenses (n = 1136).

	Weighted %(SE)		
	Complications		
	No	Yes	*p*-value
	(n = 871)	(n = 265)	
Age			0.0585
10–19 years	10.83 (1.06)	5.44 (1.51)	
20–29 years	31.62 (2.18)	28.26 (3.19)	
30–39 years	28.21 (2.08)	31.13 (3.38)	
40–49 years	20.36 (1.34)	23.79 (2.46)	
50–59 years	8.98 (1.15)	11.38 (2.08)	
Sex			**<0.0001**
Men	34.39 (2.04)	13.76 (2.44)	
Women	65.61 (2.04)	86.24 (2.44)	
Duration of contact lens use			**<0.0001**
< 1 year	34.38 (1.77)	11.82 (2.6)	
1–5 years	34.96 (1.93)	27.17 (3.33)	
5–10 years	16.66 (1.45)	24.42 (3.07)	
10 years <	13.99 (1.27)	36.59 (3.52)	
Lens type (1-month data)			
No lens use in the past month	39.73 (2.15)	47.36 (3.87)	0.0538
Soft lenses	30.72 (2.1)	34.29 (4.14)	0.4057
Hard lenses (RGP)	2.58 (0.63)	2.5 (0.91)	0.9434
Orthokeratology	0.96 (0.28)	0.34 (0.34)	0.3061
Cosmetic lens	8.03 (1.11)	15.46 (3.3)	**0.006**
Other	0.13 (0.13)	-	-
Place of purchase			-
Eye clinics	7.07 (0.94)	5.73 (1.44)	
Eyeglass stores	83.05 (1.5)	86.49 (2.34)	
Unregulated lens sellers	9.24 (1.3)	7.78 (1.98)	
Do not know	0.65 (0.29)	-	
Whether contact lenses are prescribed by an ECP			0.4271
Yes	20.05 (1.47)	24.25 (3.25)	
No	78.34 (1.53)	73.95 (3.38)	
Do not know	1.62 (0.58)	1.81 (0.86)	
Contact lens experience with respect to ophthalmic complications			
Do not know	-	0.9 (0.9)	-
Soft lenses	-	64.24 (3.52)	-
Hard lenses (RGP)	-	6.35 (1.38)	-
Orthokeratology	-	1.66 (0.85)	-
Cosmetic lens	-	29.4 (3.6)	-

SE, standard error

Tables 3 and 4 presents an analysis of the factors affecting contact lens use and complications by controlling for other variables through logistic regression. In addition to the previously mentioned factors, various other factors such as region, household income, and previously diagnosed eye disease were included as influencing factors. In the univariate analysis using logistic regression, older age (*p* = 0.0177), female sex (*p*<0.0001), pre-existing eye disease diagnosed by an ECP (*p* = 0.001), and duration of contact lens use (*p*<0.0001) were associated with the complication rate after contact lens use (Table 3). Additionally, among pre-existing eye diseases, we conducted additional analyses to examine the correlation between ocular surface-related conditions, specifically dry eye syndrome, and lens complication. As a result, dry eye syndrome exhibited a significant association with the complication rate (data not shown, p = 0.0303).

**Table 3:** Logistic regression of the relationship between the variables and increasing complication rate in contact lens users. Outcome: Complication, yes.

	Weighted % (SE)	Univariate		Multivariate	
		OR (95% CI)	*p*-value	OR (95% CI)	*p*-value
Age groups			0.0177		0.444
<20 years	13.05 (3.29)	1 (Ref.)		1 (Ref.)	
20–39 years	22.88 (1.82)	1.976 (1.072, 3.644)		1.372 (0.695, 2.709)	
40–59 years	26.37 (2.16)	2.377 (1.300, 4.348)		1.633 (0.746, 3.575)	
Sex			**<0.0001**		**0.0002**
Men	10.68 (1.82)	1 (Ref.)		1 (Ref.)	
Women	28.20 (1.71)	3.405 (2.209, 5.250)		2.275 (1.490, 3.471)	
Region			0.6561		0.6465
Urban	22.35 (1.93)	1 (Ref.)		1 (Ref.)	
Rural	23.67 (1.72)	1.068 (0.798, 1.430)		1.077 (0.783, 1.482)	
Household Income			**0.0335**		**0.0282**
Q1 (Lowest)	13.51 (4.34)	0.470 (0.214, 1.031)		0.476 (0.221, 1.028)	
Q2	17.94 (2.57)	0.658 (0.432, 1.000)		0.625 (0.391, 0.998)	
Q3	25.42 (2.32)	1.025 (0.717, 1.467)		1.054 (0.714, 1.555)	
Q4 (Highest)	24.95 (2.23)	1 (Ref.)		1 (Ref.)	
Pre-existing eye disease diagnosed by an ECP			**0.001**		**0.0288**
No	21.54 (1.38)	1 (Ref.)		1 (Ref.)	
Yes	35.45 (4.32)	2.021 (1.335, 3.061)		1.792 (1.063, 3.019)	
Duration of contact lens use			**< .0001**		**< .0001**
< 1 year	9.32 (2.11)	1 (Ref.)		1 (Ref.)	
1–5 years	18.85 (2.13)	2.251 (1.238, 4.095)		2.204 (1.277, 3.805)	
5–10 years	30.45 (3.66)	4.234 (2.346, 7.641)		3.460 (1.973, 6.067)	
10 years <	43.87 (3.82)	7.460 (4.051, 13.740)		5.534 (2.914, 10.51)	
Place of purchase			-		-
Eye clinics	19.49 (4.33)	1 (Ref.)		1 (ref.)	
Eyeglass stores	23.73 (1.42)	1.271 (0.711, 2.271)		1.354 (0.594, 3.087)	
Unregulated lens sellers	20.11 (4.95)	1.031 (0.456, 2.330)		0.822 (0.271, 2.494)	
Do not know	-	-		-	
Whether contact lenses are prescribed by an ECP			0.3797		0.8825
Yes	26.55 (2.98)	1 (Ref.)		1 (ref.)	
No	22.00 (1.57)	0.775 (0.533, 1.128)		0.979 (0.601, 1.596)	
Do not know	25.04 (11.65)	0.921 (0.249, 3.410)		1.305 (0.394, 4.325)	
Lens type (1-month data)			-		-
No lens use in the past month	20.69 (1.53)	1 (ref.)		1 (ref.)	
Soft lenses	23.81 (2.90)	1.172 (0.796, 1.728)		0.986 (0.614, 1.584)	
Hard lenses (RGP)	21.76 (8.34)	1.060 (0.398, 2.827)		0.908 (0.375, 2.196)	
Orthokeratology	10.74 (10.16)	0.459 (0.055, 3.830)		0.798 (0.086, 7.423)	
Cosmetic lens	32.33 (5.45)	1.822 (1.101, 3.017)		1.650 (0.829, 3.286)	
Other	-	-		-	
≥2 types	45.51 (15.43)	3.184 (0.91, 11.146)		3.258 (0.638, 16.632)	

SE, standard error; ORs, odds ratio; CIs, confidence intervals. *The p-value was calculated using hierarchical multivariate logistic regression analyses

**Table 4:** Logistic regression of the relationship between variables and increasing soft lenses use rate in contact lens users. Outcome: Soft lenses, use.

	Weighted % (SE)	Univariate		Multivariate	
		OR (95% C.I)	*p*-value	OR (95% C.I)	*p*-value
Age groups			**<0.0001**		**<0.0001**
<20 years	37.64 (4.88)	1 (Ref.)		1 (Ref.)	
20–39 years	37.15 (2.93)	0.979 (0.608, 1.576)		0.687 (0.395, 1.193)	
40–59 years	18.71 (2.22)	0.377 (0.220, 0.647)		0.283 (0.158, 0.508)	
Sex			0.4701		0.0048
Male	33.51 (3.41)	1 (Ref.)		1 (Ref.)	
Female	30.71 (2.11)	0.889 (0.645, 1.225)		0.590 (0.410, 0.849)	
Region			0.1359		0.1514
Urban	34.42 (2.91)	1 (Ref.)		1 (Ref.)	
Rural	28.62 (2.66)	0.758 (0.526, 1.092)		0.753 (0.510, 1.111)	
Household Income			0.9537		0.9167
Q1 (Lowest)	35.33 (9.49)	1.166 (0.500, 2.718)		1.289 (0.529, 3.142)	
Q2	30.45 (3.74)	0.934 (0.625, 1.396)		1.032 (0.663, 1.606)	
Q3	31.09 (2.89)	0.962 (0.711, 1.303)		1.083 (0.787, 1.492)	
Q4 (Highest)	31.91 (2.49)	1 (Ref.)		1 (Ref.)	
Pre-existing eye disease diagnosed by an ECP			**0.0004**		**0.0011**
No	33.56 (2.11)	1 (Ref.)		1 (Ref.)	
Yes	14.39 (3.69)	0.335 (0.184, 0.608)		0.353 (0.191, 0.655)	
Duration of contact lens use			**<0.0001**		**<0.0001**
< 1 year	22.04 (2.91)	1 (Ref.)		1 (Ref.)	
1–5 years	28.94 (3.08)	1.434 (0.976, 2.108)		1.498 (1.002, 2.240)	
5–10 years	34.6 (4)	1.859 (1.188, 2.908)		2.319 (1.420, 3.789)	
≤10 years	47.54 (3.73)	3.145 (2.090, 4.732)		5.070 (3.160, 8.134)	
Place of purchase			**0.0002**		**<0.0001**
Eyeclinics	8.09 (2.87)	1 (Ref.)		1 (Ref.)	
Eyeglass stores	33.92 (2.26)	5.781 (2.686, 12.445)		9.477 (3.773, 23.799)	
Unregulated lens sellers	27.37 (4.63)	4.247 (1.802, 10.009)		4.539 (1.566, 13.159)	
Do not know	23.02 (19.97)	3.370 (0.318, 35.665)		8.377 (0.810, 86.633)	
Whether contact lenses are prescribed by an ECP			0.9474		0.8859
Yes	31.53 (3.47)	1 (Ref.)		1 (Ref.)	
No	31.64 (2.17)	1.001 (0.706, 1.418)		0.899 (0.582, 1.389)	
Do not know	27.15 (13.17)	0.807 (0.214, 3.040)		0.965 (0.233, 4.005)	

SE, standard error; ORs, odds ratio; Cis, confidence intervals. *The *p*-value was calculated using hierarchical multivariate logistic regression analyses.

In contrast, multivariate analysis revealed a significant relationship between the complication rate and female sex (*p* = 0.002), pre-existing eye disease diagnosed by an ECP (*p* = 0.0288), and duration of contact lens use (*p*<0.0001) among contact lens users (Table 3). No other variables were identified as risk factors associated with an increased complication rate among contact lens users. In the univariate analysis for soft lens usage, the 20–39-year age group (*p*<0.0001), non-pre-existing eye disease diagnosed by an ECP (*p* = 0.004), duration of contact lens use (*p*<0.0001), and place of purchase (*p* = 0.0002) were associated with soft lenses usage (Table 4). No other factors were significantly associated with soft lens usage. In contrast, multivariate analysis revealed a significant relationship between soft lens usage and the 20–39-year age group (*p*<0.0001), male sex (*p* = 0.0048), non-pre-existing eye disease diagnosed by an ECP (*p* = 0.0011), duration of contact lens use (*p*<0.0001), and place of purchase (*p*<0.0001) in contact lens users (Table 4). No other variables were identified as risk factors for increased soft lens usage.

## Discussion

With an increasing number of contact lens users in South Korea, research on contact lens usage trends is being consistently conducted. A multi-institutional, nationwide survey on contact lens use was conducted from October 2000 to September 2002 with 482 individuals [19], and a similar study involving 920 high school students in a specific region was published in 2011 [20]. To the best of our knowledge, this is the first large population-based study to investigate trends in contact lens use in South Korea.

Considering the advancements in medical technology and the improvement of socioeconomic conditions, this study presents a comprehensive report on the status and complication trends of contact lens use on a nationwide scale, encompassing individuals of all ages, with a sample size of 1,136 cases. In addition, while previous studies were limited to analyzing contact lens usage in a specific cohort of contact lens wearers, this study evaluated the proportion of contact lens users in the entire population, providing a more accurate understanding of trends in contact lens use.

The most notable observation from this study was the increase in the age of contact lens users and the decrease in the rate of female users compared to the data from previous studies.

We indirectly compared our study with the report published in 2004, which investigated contact lens user trends using questionnaires distributed in universities and hospitals [19]. Although the previous study was not a population-based study, we were able to present approximate changes in contact lens usage patterns. According to that report [19], the main contact lens users were the 20–29-year age group, accounting for 72% of the total sample, followed by the 30–39-year group at 16.8% and ≥40 years old at only 2.7%. In the present study, however, the average age of contact lens users was 33.42 years; 28.88% were aged 30–39, and 30.7% were aged ≥40 years, indicating that the average age of contact lens users is increasing. Further, women accounted for 70.36% of our sample, showing a reduction from 88% reported in a 2004 study [19]. We compared our findings with the 2020 Global Contact Lens Prescribing Report by Morgan [5]. Morgan’s study comprises a much larger sample size, having collected data from prescribing practitioners and encompassing over 400,000 contact lens fits from 71 countries. The study’s primary objective was to document global trends in contact lens prescriptions; thus, it focused on a limited number of parameters, including material, modality, frequency of wear, type of correction, and care system. In contrast, our study involved a small number of lens users, approximately 3,000, who voluntarily selected their lens type, and were from a single national study. Furthermore, our study gathered various data types, such as lens type, duration of lens wear, places of lens purchase, and the presence of complications. Despite these differences, we found it valuable to compare the two studies to gain insights into the general similarities and differences in lens-wearing patterns.

In 2020, a multi-institutional survey was conducted with 13,311 individuals aged 1–75 years across 24 countries. The average age of the participants was 32.4±15.6 years, similar to that in our study. However, the proportion of women contact lens users was 65%—lower than that reported in our study (70.36%), indicating that a large proportion of young women are using contact lenses for cosmetic purposes and that there are more middle-aged contact lens users than in the past. This is probably because middle-aged women started using contact lenses during the 2000s when they were in their 20s and have continued to wear them since.

Figs 2 and 3 present the purpose of lenses used in South Korea by comparing the regional lens user ratio and the percentage of lens types currently in use. Fig 2B and 2C present the percentages of users aged 20–39 years and women users, who were the majority of lens users in previous studies [16, 19, 20], stratified by region. The contact lens usage rates in Seoul, Gwangju, Busan, and Daejeon were similar. The percentage of contact lens use experience in the past one month was 48% for soft lenses, 33% for cosmetic lenses, 10% for OK lenses, and 4% for RGP lenses (Fig 3).

We further compared the lens usage trends of our study with those in Japan, a country with similar ethnic characteristics where the primary purpose of contact lens use is cosmetic enhancement. In a study by Itoi *et al*. [10] conducted at Kyoto Medical University, Japan, contact lens users aged 16–60 years were surveyed between 2003 and 2016; the prevalence of cosmetic lens use was ≤15% during the study period, lower than the prevalence in South Korea (33%). This indicates that the use of cosmetic lenses in South Korea is high, especially among the young female population.

We analyzed the contact lens usage patterns separately for men and women. We found distinct differences in usage patterns between the two sexes, which, to our knowledge, have not been reported previously. Teenage women were more likely to be contact lens users (*p* = 0.1379), significantly more likely to wear lenses for long periods of time (*p*<0.0001), and significantly more likely to use cosmetic lenses than men (*p*<0.0001) (Table 1).

Additionally, we analyzed the contact lens-related complications based on population factors. Due to limitations on the number of survey questions, we were unable to include inquiries about specific types and severity of complications, as well as lens modality. Therefore, the information presented regarding contact lens complications should be viewed with caution. However, this study is significant as it is one of the first attempts to explore trends in contact lens complications within the Korean population, considering the lack of population-based research on this subject in Korea.

Therefore, our study analyzed the impact of various population-based factors on lens-related complications. Incidences of contact lens complications were significantly more common in women (*p*<0.0001), although complications from soft lenses were significantly more common in men than in women *(p* = 0.0151) (Table 1). Table 2 shows that female contact lens wearers had a significantly higher frequency of complications than males (*p*<0.0001). Most female contact lens users in South Korea have been wearing lenses for at least five years (46.45%), as shown in Table 1. This can explain the high rate of complications among female contact lens users.

More than 50% of male contact lens users had been wearing contact lenses for less than a year, and within the contact lens user group, men preferred soft lenses. Complications occurred even after a short period of use, usually less than one year, suggesting that insufficient care may also be a contributing factor.

We conducted a multivariate analysis to identify factors that were strongly correlated with complications after contact lens use and found that the percentage of complications among women was higher even after adjusting the duration of contact lens use. This suggests other potential contributing factors, such as make-up, a high prevalence of cosmetic lens use, or a lower threshold for discomfort among women. This finding aligns with the results of a study conducted by Kim in 2008–2009 [16], where a significant increase in complications was observed among teenage patients using cosmetic-colored contact lenses, with a prevalence of 32.33%. The percentage of complications also increased with household income, but this may be due to the proportional relationship between income and medical services. Instead, the correlation between income and complications may have been underestimated owing to the tendency of the lower-income group to have a lower level of medical use.

The group diagnosed with ophthalmic diseases by an ECP showed 1.8-fold higher rates of complications than the other groups (Table 3), indicating the correlation between underlying ophthalmic diseases and lens use complication. Therefore, individuals with ophthalmic diseases require proper care from an ECP to prevent complications while using contact lenses. However, our study showed that over 80% of contact lens users purchased lenses from eyeglass stores without prescription, which means that their underlying complications may have not been addressed before using contact lenses (Table 1). Given the increased risk of ocular diseases such as macular degeneration, cataracts, and glaucoma in individuals aged over 40 years, it is crucial to enhance patient education about the significance of from ECP before contact lens use.

As this was a cross-sectional study, limitations such as the risk of recall or information bias cannot be denied. In addition, we could not determine the causal relationship between the variables and the rate of complications because we investigated the odds ratios rather than the hazard ratios. Unfortunately, the KNHANES is a survey that investigates various health conditions and nutritional status, not limited solely to ophthalmic examinations. Due to the constraints on the number of survey questions for specific conditions, questions related to various complication types and severity and lens modality were not covered in the survey. Contact lens modality and types of lens care solutions are critical factors that can impact lens use complications; however, due to the reasons mentioned above, these aspects were not investigated in the present study. Therefore, conducting in-depth research on the specific risk factors that may be related to critical complications to focus on and provide care in these specific areas is warranted. Finally, questions related to contact lenses were included in the KNHNES only from 2020 to 2022. Only the 2021 survey included all age groups; the 10–39-year age groups were not included in the 2020 or 2022 surveys. Therefore, it would be beneficial to conduct further research with longer study durations to discover trends over longer periods of time.

Despite these limitations, the significance of this study cannot be overlooked. Foremost, this is the first study on contact lens usage in a uniform population of a single ethnicity, country, and at a single research institute. The advantages of conducting epidemiological research within a single ethnicity are notable. This approach enhances internal consistency, allowing for more coherent and reliable findings. Furthermore, it provides valuable insights into the population’s specific characteristics, sensitivities, and unique healthcare needs, which can inform future research and individualized patient care. Second, this study is the largest and latest of its kind conducted in South Korea. The sample size of this study was 1,136, the largest population in a single country covered in a study since 2016. Additionally, this is the latest large-scale study of contact lens usage trends in South Korea since 2014, as Korean national data have only recently become available. In addition, trends in lens use can be studied precisely by directly identifying the percentage of contact lens users within the entire population.

## Conclusion

It has been more than 30 years since individuals in South Korea began wearing contact lenses. The average age of lens users has increased since then. The frequency of wearing lenses for aesthetic purposes among women in South Korea is still much higher than that in other countries. Nevertheless, the demand for multifocal soft contact lenses among middle-aged and elderly individuals is expected to increase for myopic regression, considering the high rate of myopia in the East Asian population. To ensure safe contact lens use, it is advisable to emphasize the significance of precise prescriptions, screening for eye conditions, and guidance from eye care professionals.
